# Antiphase Boundaries as Faceted Metallic Wires in 2D Transition Metal Dichalcogenides

**DOI:** 10.1002/advs.202000788

**Published:** 2020-06-08

**Authors:** Jung Hwa Kim, Se‐Yang Kim, Sung O. Park, Gwan Yeong Jung, Seunguk Song, Ahrum Sohn, Sang‐Woo Kim, Sang Kyu Kwak, Soon‐Yong Kwon, Zonghoon Lee

**Affiliations:** ^1^ School of Materials Science and Engineering Ulsan National Institute of Science and Technology (UNIST) Ulsan 44919 Republic of Korea; ^2^ Center for Multidimensional Carbon Materials Institute for Basic Science (IBS) Ulsan 44919 Republic of Korea; ^3^ Department of Energy Engineering School of Energy and Chemical Engineering Ulsan National Institute of Science and Technology (UNIST) Ulsan 44919 Republic of Korea; ^4^ School of Advanced Materials Science and Engineering Sungkyunkwan University (SKKU) Suwon 16419 Republic of Korea

**Keywords:** anisotropy, antiphase boundary, faceted line defects, in‐plane mobility, WS_2_/graphene heterostructures

## Abstract

Antiphase boundaries (APBs) in 2D transition metal dichalcogenides have attracted wide interest as 1D metallic wires embedded in a semiconducting matrix, which could be exploited in fully 2D‐integrated circuits. Here, the anisotropic morphologies of APBs (i.e., linear and saw‐toothed APBs) in the nanoscale are investigated. The experimental and computational results show that despite their anisotropic nanoscale morphologies, all APBs adopt a predominantly chalcogen‐oriented dense structure to maintain the energetically most stable atomic configuration. Moreover, the effect of the nanoscale morphology of an APB on electron transport from two‐probe field effect transistor measurements is investigated. A saw‐toothed APB has a considerably lower electron mobility than a linear APB, indicating that kinks between facets are the main factors of scattering. The observations contribute to the systematical understanding of the faceted APBs and its impact on electrical transport behavior and it could potentially extend the applications of 2D materials through defect engineering to achieve the desired properties.

## Graphical abstract

Herein, the anisotropic nanoscale features of antiphase boundaries in 2H phase of transition metal dichalcogenides are investigated (i.e., saw‐toothed for transition metal‐facing and linear for chalcogen‐facing antiphase boundaries) in order to preserve the thermodynamically most stable atomic configurations. In addition, they have different in‐plane transport behavior because lots of kinks in the saw‐toothed antiphase boundary act as scattering centers.

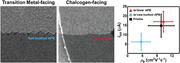

Following the isolation and characterization of graphene, 2D transition‐metal dichalcogenides (TMDs) with intrinsic bandgaps have attracted extensive attention, principally because of the many way by which their electronic and chemical properties can be modulated including i) the possibility of different stable phases^[^ ^1^, ^2^ ^]^ and ii) the presence of different types of structural defects.^[^ ^3^, ^4^ ^]^ For example, point defects (e.g., vacancy, adatoms, dopants, etc.) induce discrete gap states, resulting in nonzero electronic states within the bandgap.^[^ ^3^, ^5^, ^6^, ^7^ ^]^ Likewise, a grain boundary (GB), which is a type of 1D defect in 2D MoS_2_, generates a defect state from the Mo 4d orbital at the 5–7 dislocation cores,^[^ ^8^, ^9^ ^]^ and causes local variation of bandgap at the boundary.^[^ ^10^ ^]^ In addition, antiphase boundary (APB), which is a special GB between two crystals having the same crystallographic orientation but are phase‐shifted by 60° with respect to each other, creates dispersive mid‐gap states across the Fermi level, similar to 1D metallic nanowires embedded in a 2D semiconductor matrix,^[^ ^11^, ^12^, ^13^, ^14^, ^15^, ^16^ ^]^ such a structure could be potentially applied in a fully 2D‐integrated circuit.^[^ ^11^ ^]^ In the case of TMDs with a trigonal prismatic (2H) phase, conductive 1D APBs can be exploited in several ways. First, electron beam (e‐beam) irradiation in transmission electron microscopy (TEM) manipulates the dense networks of APBs from the chalcogen‐deficient environment^[^ ^17^, ^18^ ^]^ due to the low knock‐on threshold voltage of chalcogen atoms in TMDs (e.g., S for 80 kV, Mo for 560 kV^[^ ^19^, ^20^ ^]^). Second, epitaxial growth of TMDs on a selected substrate (e.g., sapphire,^[^ ^21^, ^22^ ^]^ graphene,^[^ ^23^, ^24^ ^]^ hexagonal boron nitride,^[^ ^25^ ^]^ etc.) is a facile method for manipulation of high probability of APBs, because of the confined lattice orientation. Various energetically stable APB structures have been described depending on which elements are present face to face across the boundary (i.e., transition‐metal‐facing and chalcogen‐facing),^[^ ^11^ ^]^ but in all previous experimental reports,^[^ ^15^, ^22^, ^26^, ^27^ ^]^ a dense network of chalcogen‐facing APBs has been mainly observed.

Faceted GBs are commonly observed in bulk metals and semiconductors and are associated with transition to lower energy states than the corresponding original GBs, despite the increased area.^[^ ^28^ ^]^ In a 2D system, APBs form nanometer scale facets of ±20° relative to the zigzag (ZZ) direction, due to the armchair (AC) edge effect, which is another stable edge state.^[^ ^15^, ^29^ ^]^ Herein, we report anisotropic nanoscale morphologies of APBs depending on what elements are encountered in the single‐layer WS_2_ using the multimode of TEM. WS_2_ flakes were directly grown on a graphene substrate so as to have a high probability of APBs through van der Waals epitaxy. Interestingly, we find that S‐facing and W‐facing APBs exhibit different nanoscale morphologies (i.e., linear for S‐facing APBs, saw‐toothed for W‐facing APBs), although both of these APBs have the same atomic configuration, consisting of over‐ or under‐coordinated S atoms. We have confirmed experimental results through density functional theory (DFT) calculations to determine the energetically stable atomic configurations of APBs. Conductive atomic force microscopy (C‐AFM) results show that both types of APBs behave as metallic wires due to the same 4‐ and 8‐membered atomic configurations,^[^ ^11^, ^15^ ^]^ but the two‐probe field effect transistor device (FET) results show reduced electron mobility in saw‐toothed (highly faceted) APBs, indicating that the kinks between facets could be a dominant source of electron scattering. Our results contribute to a comprehensive understanding of nanoscale faceted 1D defect and its effect on transport behavior.

We synthesized single‐layer WS_2_ flakes on a graphene substrate by a chemical vapor deposition (CVD) (see the Experimental Section for more details on the experiment). **Figure** **1**a,b shows the dark‐field TEM (DF‐TEM) image and selected‐area electron diffraction (SAED) result of a single‐crystal WS_2_ flake grown on graphene, respectively. Because of the threefold symmetry of WS_2_, the six [$\bar{1}100$] diffraction spots (white‐shaded) denoted as *k*_W_ and *k*_S_, indicating tungsten‐ and sulfur‐related planes, respectively. Thus, the intensity profile in Figure 1b shows different intensities for *k*_W_ and *k*_S_ due to the different atomic form factors of tungsten and sulfur.^[^ ^15^ ^]^ From the DF‐TEM and SAED analysis, we estimate that the WS_2_ flakes are terminated by a W‐edge under our growth conditions (Figure 1a,b and Figure S1, Supporting Information). In addition, WS_2_ diffraction spots (*a* = 0.272 nm, white‐shaded circle) are well‐aligned with the graphene diffraction spots (*a* = 0.213 nm, green‐shaded circle), indicating that the WS_2_ flakes grow epitaxially on graphene along the most energetically preferred orientation.^[^ ^23^, ^24^ ^]^ A larger view of the sample is seen in the scanning electron microscopy (SEM) image in Figure 1c that shows ribbon‐shaped WS_2_ flakes formed by merging two inversion domains due to the confined nucleation orientation via vdW epitaxy. When flakes join, GBs are invariably created and especially, GBs between flakes with 60° phase shift, called APBs, are formed. AFM height analysis (Figure 1d) shows that the merged flakes have a uniform monolayer thickness of ≈0.7 nm through the APB.

**Figure 1:**
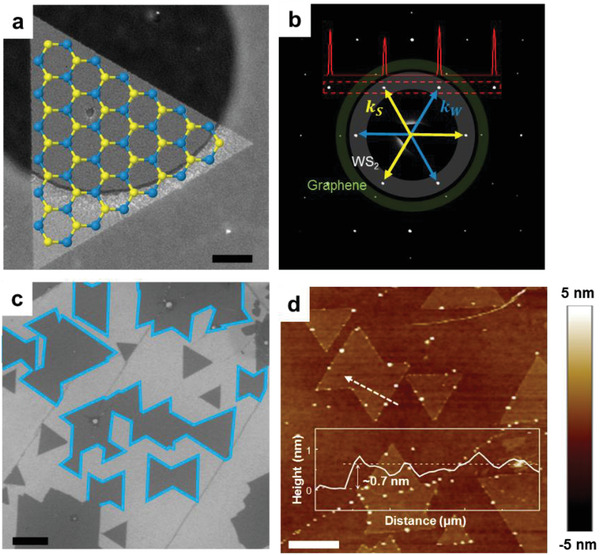
Antiphase boundaries (APBs) of WS_2_. a) DF‐TEM image and b) Diffraction pattern of a WS_2_ flake directly grown on graphene substrate. S and W sublattices induce two different intensities of the [$\bar{1}100]$ diffraction spots (inset), represented by *k*_S_ and *k*_W_. c) SEM image of WS_2_ grown on graphene. WS_2_ flakes have a specific orientation due to the epitaxial growth of WS_2_ on the graphene substrate, resulting in the formation of APBs rather than tilted GBs. d) AFM height image of a merged WS_2_ flake and its line profile across the APB. The scale bars for (a) and (c,d) are 200 and 500 nm, respectively.

To investigate the nano‐ and atomic‐scale structures of the APBs, DF‐TEM and high‐angle annular dark‐field scanning TEM (HAADF‐STEM) analysis were performed as shown in **Figure** **2**. DF‐TEM results in Figure 2a,b,d,e were obtained using the adjacent [$\bar{1}100$] spots (SAEDs given in the insets in Figure 2a,d), representing red‐square regions in the bright‐field TEM (BF‐TEM) images (insets in Figure 2b,e). Despite the fact that there is only one set of diffraction spots in the SAED pattern, DF‐TEM results indicate the presence of two grains joined through an inevitable APB. Note that the possibility of layer boundary could be excluded, due to the uniform intensity in BF‐TEM images. From the difference in DF‐TEM intensities at *k*_W_ and *k*_S_ in the SAED pattern (Figure 1b), the exact orientation of each grain can be determined. It is striking that the W‐facing APB in Figure 2a,b has a saw‐tooth shape with high density of facets, while the S‐facing APB in Figure 2c,d shows a linear shape in nanoscale. Figure 2c,f shows the local directionality of the W‐facing and S‐facing APBs using the HAADF‐STEM, obtained from the black‐dotted rectangular region in Figure 2b,e, respectively. In the local view of the S‐facing APB, it is seen that its direction (Figure 2f) is rotated by ≈17° from the S‐ZZ direction, which is in accordance with results from previous reports.^[^ ^15^, ^22^ ^]^ Likewise, in the W‐facing APB, each facet is rotated by around 20° from the S‐ZZ, showing a slightly higher angle than the S‐facing. The effect of the AC edge could determine the inclination angle of the APB facet from the ZZ edge direction ^[^ ^29^ ^]^ (Figure S2, Supporting Information). Facets in the W‐facing APB have the <$\bar{23}10$> directions, which are the closest to the AC edge direction. Insets in Figure 2c,f are Fourier‐filtered images obtained after removing crystal information of WS_2_, and a peculiar contrast is seen at the APB. It could be explained by symmetry interruption at APB, which would affect to a broken‐symmetry‐driven charge redistribution.^[^ ^30^ ^]^ This indicates that the electronic characteristics are modified along the APB when compared to the rest of the semiconducting WS_2_ matrix. For MoS_2_ films grown on sapphire by physical vapor deposition (PVD) (Figure S3, Supporting Information), it is seen that the transition‐metal‐facing (M‐facing) APBs generally have a saw‐toothed shape, while chalcogen‐facing (X‐facing) APBs are linear. To sum up, regardless of the substrate, growth method, and the TMD type, APBs in all TMDs have an anisotropic nanoscale structure, depending on which elements they face at APB.

**Figure 2:**
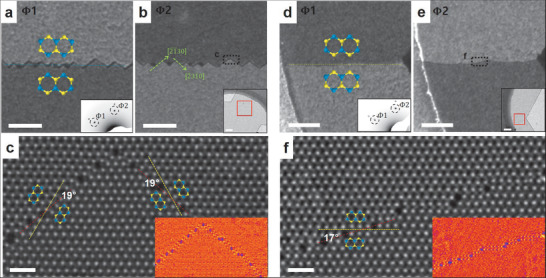
Unusual overall structure of APBs in monolayer WS_2_ depending on whether they are W‐facing or S‐facing. a,b,d,e) DF‐TEM images of W‐facing and S‐facing APBs, respectively. Reverse contrast between Φ1 and Φ2 images showing the presence of anti‐phase between the two grains. Insets in (a,d) and (b,e) are the diffraction patterns and bright‐field images, respectively. The overall structure of each APB has either a saw‐toothed (W‐facing) or a linear (S‐facing) shape. (c,f) AR‐STEM images of saw‐toothed and linear APB, respectively. Locally, W‐facing and S‐facing APBs have the same atomic structure. Each facet is rotated by around 20° from the S ZZ direction.^[^ ^15^ ^]^ Blue (a) and yellow (c,d,f) dotted lines indicate W and S ZZ directions, respectively. Red lines in (c,f) show the facet directions of each of these APBs. Scale bar, (a,b,d,e) and (c,f) 50 and 1 nm, respectively.

For comparison between M‐facing and X‐facing APB, **Figure** **3** describes how two inverted domains meet and merge on a domain‐scale and a nanoscale, respectively. Figure 3a shows DF‐TEM image of asymmetrically merged flake and schematics of APB formation. The inner triangle is the nucleation site of WS_2_ with W‐ZZ edge terminations and sequential triangles depicted as thin lines represent laterally growing grains at a constant time interval. When inverted grains meet and continue to grow, two types of straight boundary (Straight boundaries for both cases in domain‐scale depicted in Figure S12b, Supporting Information) are expected to be formed following the growth kinetics: along parallel to the W‐ZZ line (W‐facing) or along intersections of joint edges of same growth level (S‐facing).^[^ ^31^ ^]^ However, nanoscale view using DF‐TEM image shows that the domain‐scale straight boundary could be categorized as saw‐toothed and linear morphology on nanoscale dimension depending on the element‐facing. Especially, the W‐facing APB has a highly faceted structure on nanoscale. For detailed comparison between two types of APBs, statistics analysis on facet length and facet inclination angle were performed using many numbers of DF‐TEM images (>45 total images) as shown in Figure 3b,c. While the average facet length of W‐facing APBs is a few nanometers, S‐facing APBs are up to hundreds of nanometers with large standard deviations because they are highly dependent on the size of the grown flakes. In addition, the facets in the W‐facing APBs have closer to the AC edge direction compared to the S‐facing APBs, which means the W‐facing APBs are more affected by the AC edge, consistent with Figure 2c. Averaged values of APB facet length and inclination angle from S‐ZZ line are described in Table S1 (Supporting Information). To unravel the discrepancy in nanoscale morphology of APBs, we studied on stable atomic configurations that could be explained by thermodynamics.

**Figure 3:**
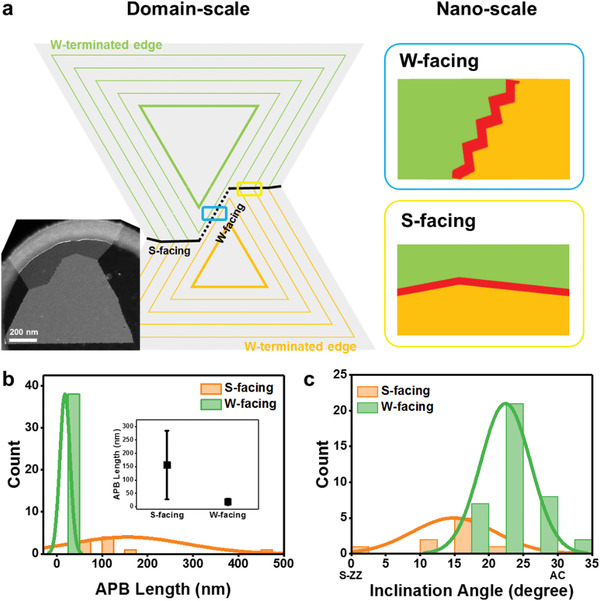
Comparison of two types of APBs. a) DF‐TEM image and corresponding schematics of domain‐ (left) and nanoscale (right) W‐facing and S‐facing APBs. Green and orange sequential triangles represent laterally growing grains with W‐terminated. b,c) Statistical data showing APB facet length (b) and inclination angle from the S‐ZZ (c) of W‐facing and S‐facing APBs, respectively.

As mentioned above, APBs can be categorized into two groups according to the front‐facing element (i.e., S‐ and W‐facing). Depending on how the unit cells of each grain meet, each group can be divided into two representative cases (i.e., having a 4‐membered or 8‐membered configuration at the APB). Therefore, as shown in Figure **4**a–d, we consider the four possible APB structures, denoted by W‐44 (4a), W‐88 (4b), S‐44 (4c), and S‐88 (4d). Here, the letter W(S) denotes a W‐(S‐) facing APBs, and 4(8) is the number of atoms composing one unit of the APB. While the considered configurations have a mirror plane at the APB in our calculations, experimental results (Figures 2c,f and 4e) show untypical structures. Therefore, we introduce the unit configuration of APBs, and each unit of W‐44, W‐88, S‐44, and S‐88 is denoted as W_rhomb_, W‐S‐W, S_rhomb_, S‐W‐S, respectively. Figure 4e shows the atomic‐scale structure of a W‐facing saw‐toothed APB imaged by atomic‐resolution TEM (AR‐TEM). We determine the exact orientation of each WS_2_ grain using the S vacancy (yellow arrow in Figure 4e), which is easily formed under e‐beam irradiation.^[^ ^19^ ^]^ Each facet is rotated by around 22° from the S‐ZZ line and is made up of S_rhomb_ and W‐S‐W, identical to the atomic configuration of the S‐facing APB in Figure 2f. To determine why identical atomic configurations of APBs (rhombohedral‐shaped S‐facing APB and linearly‐connected W‐facing APB) originate from varying nanoscale structures, we have evaluated the structural stability of these APB structures by DFT calculations (Figure S4 and Note S1 in the Supporting Information for detailed explanation). Note that for all APB models, the edges are constrained at 50% and 100% coverage of S atoms, exhibiting the most stable configurations for W‐ and S‐edges, which were determined from edge formation energy (*γ*_edge_) calculations (Figure S5, Supporting Information). W‐edges are the most stable for a whole range of sulfur chemical potentials (∆*μ*_S_), which is in accordance with our experimental result (Figure S1, Supporting Information). The APB formation energy (*γ*_APB_) results show that S‐44 and W‐88 APBs are more stable than those of the S‐88 and W‐44 APBs over the whole range of ∆*μ*_S_ (Figure 4f) and relaxed structures by DFT calculations are represented in Figure S6 (Supporting Information). In particular, it was found that the combined S_rhomb_ and W‐S‐W APB (i.e., S_rhomb_ + W‐S‐W) is more stable than ideal W‐S‐W APB, indicating that W‐S‐W APB is preferred to coexist with S_rhomb_ regardless of ∆*μ*_S_ (Figure S7, Supporting Information). Compared to the bulk WS_2_, S‐44 and W‐88 APBs consist of over‐ and under‐coordinated of S atoms, respectively, while S‐88 and W‐44 APBs contained the over‐ or under‐coordination of W atoms, respectively, (Figure S8, Supporting Information). Accordingly, rearrangements in W‐coordination in APBs are expected to cause higher endothermicity than those in S‐coordination. Next, we investigated the structural transition between WS_2_ APBs, which might be caused by the sliding of one grain along the APB line (Figure S9, Supporting Information). By checking the endothermicity of the reverse reactions, we predicted that the transition might occur only in one direction for W‐facing APBs (i.e., from W‐44 to W‐88) and S‐facing APBs (i.e., from S‐88 to S‐44) with small and negligible energy barriers, respectively. Finally, S‐44 and W‐88 APBs are energetically more favorable than S‐88 and W‐44 APBs, respectively. Considering that the S‐44 is found to be more stable than W‐88 for the wide range of ∆*μ*_S_, APBs are expected to have S_rhomb_ predominantly, followed by W‐S‐W, which agrees with experimental statistics (Figure 4g). We observe that for both W‐ and S‐facing APBs, although the ratio is different, S_rhomb_ configurations are dominant, followed by W‐S‐W.

**Figure 4:**
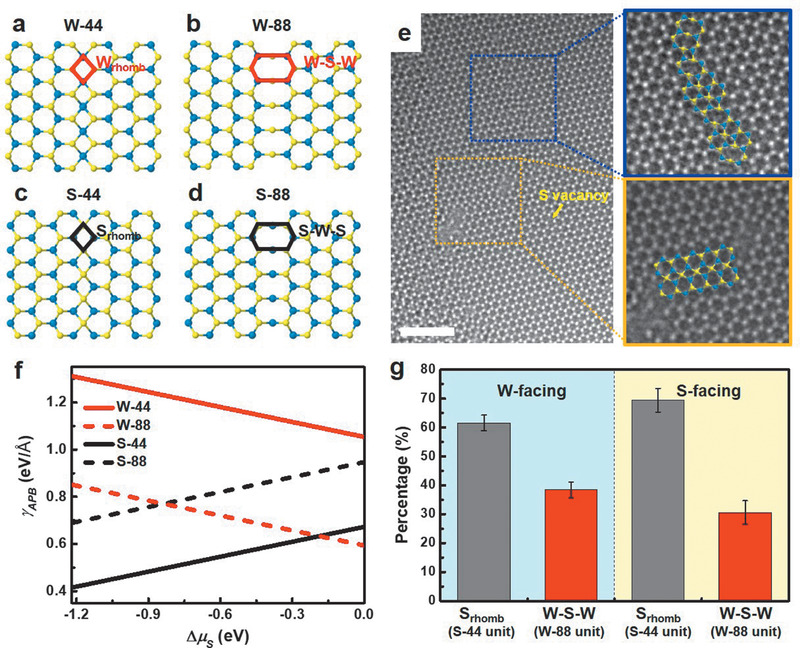
Atomic configuration of APBs. a–d) Atomistic configurations of the different plausible WS_2_ APB models, including the rhombohedral W‐facing APB (denoted as a) “W‐44”), elongated hexagonal W‐facing APB including 8 atoms (denoted as b) “W‐88”), rhombohedral S‐facing APB (denoted as c) “S‐44”), and the elongated hexagonal S‐facing APB including 8 atoms (denoted as d) “S‐88”). Units of each of these APBs are denoted as a) “W_rhomb,_” b) “W‐S‐W,” c) “S_rhomb_,” and d) “S‐W‐S.” e) AR‐TEM image of a W‐facing APB including mainly S_rhomb_ and some of W‐S‐W. f) APB formation energy (*γ*_APB_) of S‐facing and W‐facing APBs as a function of the chemical potential of S (∆*µ*_S_). WS_2_ becomes stable within the range of −1.22 eV < ∆*µ*_S_ < 0 eV where the upper and lower limits correspond to S‐rich and S‐poor conditions, respectively. g) Distribution of S_rhomb_ and W‐S‐W units in APBs; S_rhomb_ units are predominant, followed by W‐S‐W units. Scale bar for (e) 2 nm.

Note that highly faceted W‐facing APB (composing mixed S_rhomb_ and W‐S‐W configurations) increase the length by 28.16% compared to the expected straight W‐facing APB (composing W‐S‐W configuration alone) as depicted in Figure S10 (Supporting Information). Although faceted morphology increases total length, faceted APB including thermodynamically stable mixed configurations has lower energy state in S‐poor condition than the assumed straight W‐facing APB following the total energy calculation, which is described in detail in Note S1 and Figure S10 (Supporting Information). To sum up, we can conclude that the predominant observation of faceted APB by the DF‐TEM (Figure 2b) and AR‐TEM (Figure 4e) is related to the thermodynamic preference based on total energy evaluation, particularly under the S‐poor condition.

It is well known that all APB configurations have metallic properties due to dispersive midgap states.^[^ ^11^ ^]^ Metallicity at an APB is commonly observed by scanning tunneling microscopy (STM),^[^ ^16^, ^32^, ^33^ ^]^ due to the confined dimension of the APB (around a few nanometer width). To evaluate out‐of‐plane charge transport at the APB, we performed C‐AFM measurements using the WS_2_/graphene heterostructure. Graphene served as the back electrode and a Pt‐Ir‐coated conductive tip was used to measure current as a function of the *x–y* position in contact mode.^[^ ^34^ ^]^ The height image of a monolayer of WS_2_ including S‐ and W‐facing APBs on graphene (**Figure** **5**a) shows no contrast at APB, but the simultaneously recorded current image shows a different contrast at the APB as shown in Figure 5b. WS_2_ flakes have lower current than the surrounding graphene, while enhanced electrical conductance was observed along the APB, indicating 1D metallicity at S‐ and W‐facing APBs having the S_rhomb_ and W‐S‐W atomic configurations.

**Figure 5:**
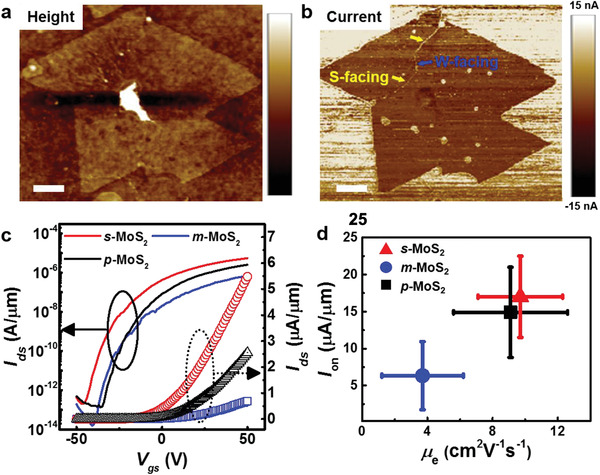
Electronic properties of APBs. a,b) Height and current AFM images of APBs in WS_2_ grown on a graphene substrate, showing metallic property along the APBs. c) Logarithmic and linear scale transfer curves (*I*_ds_–*V*_gs_) at *V*_ds_ = 1.0 V and d) On‐current (*I*_on_) versus mobility (*µ*_FET_) plots of *p*‐MoS_2_, *s*‐MoS_2_, and *m*‐MoS_2_ devices. Scale bar for (a,b) 200 nm.

To study the collective effect of saw‐toothed APB on the electrical performance, we fabricated a large number (>30) of FET devices of pristine MoS_2_ flakes (*p*‐MoS_2_), and MoS_2_ with S‐ (*s*‐MoS_2_) and Mo‐facing APBs (*m*‐MoS_2_). To fabricate FET devices and measure the transport, we used large‐area MoS_2_, another representative TMD material, grown on SiO_2_, a typical dielectric layer. All the different types of devices were patterned into ribbons with 5 µm channel length and 20 µm channel width (Figure S11, Supporting Information). The fabricated devices are depicted in Figure S12 (Supporting Information). Figure 5d shows the logarithmic (solid lines) and linear (open curves) scale transfer curve of *p*‐MoS_2_, *s*‐MoS_2_, and *m*‐MoS_2_ devices at room temperature under vacuum (<10^−3 ^Torr). The averaged on‐state current (*I*_on_) of the *s*‐MoS_2_ devices at *V*_ds_ = 1 V is ≈ 3.4 µA µm^−1^, which is almost three times higher than that of the *m*‐MoS_2_ devices (≈ 1.27 µA µm^−1^), although both APBs exhibit metallicity and have identical atomic configurations. In addition, the field effect mobility (*μ*_FET_) was calculated, according to the following Equation (1)^[^ ^35^ ^]^

(1) $$\begin{array}{c} \mu_{\mathrm{FET}}=\left(\frac{dI_{ds}}{dV_{g}}\right)\;\left[\frac{L}{WC_{i}V_{ds}}\right] \end{array}$$

where *L* and *W* are the channel length and width, respectively, and *C*_i_ is the capacitance of the SiO_2_ dielectric layer (11.5 nF cm^−2^ for 300 nm thick SiO_2_). The obtained *µ*_FET_ and *I*_on_ from 30 devices depicted in Figure S13 (Supporting Information) and summarized in Table S2 (Supporting Information). The averaged charge carrier *μ*_FET_ values are 9.7 ± 2.6 and 3.7 ± 2.5 cm^2^ V^−1^ s^−1^ for transport along *s*‐MoS_2_ and *m*‐MoS_2_ devices, respectively. To explain the difference in transport behavior between the two types of APBs, we first measured the threshold voltage (*V*_th_), which allowed to estimate the doping levels.^[^ ^35^ ^]^ However, the obtained averaged *V*_th_ for *s*‐MoS_2_, *m*‐MoS_2_, and *p*‐MoS_2_ devices are similar, indicating that doping at APB did not influence the *μ*_FET_. It is well known that the defect density (e.g., the length of APB) is closely related to the degradation of electronic properties including *µ*_FET_.^[^ ^36^ ^]^ The devices fabricated with Mo‐facing APB have a higher defect density with a longer defect line. However, while the length ratio between Mo‐facing and S‐facing APBs is around 1.88, the *I*_on_ and *µ*_FET_ ratios are significantly higher (2.68 and 2.62, respectively), indicating that there may be another parameter influencing the electron transport degradation in addition to the defect density. In addition, *s*‐MoS_2_ devices show no adverse effect on transport behavior when compared to *p*‐MoS_2_ devices,^[^ ^36^ ^]^ indicating that APB with its atomic defects does not greatly degrade electrical transport.

Previous researches reported that the W‐S‐W acts as kink^[^ ^9^ ^]^ between facets and it leads to disordered random potential wells in S_rhomb_‐based periodic potential wells for APB. According to the localization theory,^[^ ^37^ ^]^ as the density and depth of potential well is increasing, electronic wave function in the material becomes localized, which results in reduced electron transport with trapped free electrons. With the combination of increased kink density at W(Mo)‐facing APB, we suggest that the reduced *I*_on_ and *µ*_FET_ of *m*‐MoS_2_ is due to the localized electron at the random disordered potential between facets acting as intrinsic scattering centers, whereas *s*‐MoS_2_ sustained its electronic transport behavior along the channel with an atomically faceted 1D metallic wire. To sum up, it could be suggested that differences in the connection density between facets and different facet lengths lead to a significant difference in electrical properties.

In conclusion, we elucidated the anisotropic features of APBs and their different transport behavior, using representative TMDs, WS_2_ and MoS_2_. We compared two types of APBs, the transition‐metal‐facing APB and the chalcogen‐facing APB, which have saw‐toothed and linear nanoscale morphology, respectively. Using experimental and computational results, we infer that the anisotropy of the nanoscale morphology in the APB structure arises in order to preserve the most thermodynamically stable configuration, which we have denoted as S_rhomb_ and W‐S‐W. In addition, two‐probe FET measurements showed that the saw‐toothed APB has a lower electron mobility than the linear APB, because connections between facets in the saw‐tooth can act as a scattering center. Our results offer comprehensive understanding of the anisotropy of the 1D APB defect dependence on the nature of the facet, which could not be predicted using theoretical models, and the influence of the anisotropic features on the electronic transport behavior. Our study thus provides a valuable insight into nanostructure‐related electronic transport property and could extend the applications of 2D materials, where properties can be tuned through defect engineering.

## Experimental Section

##### Synthesis of WS_2_/Graphene Heterostructure

Graphene for substrate was grown on 25 µm thick Cu foils (Alfa Aesar, 99.8% purity) using a hot‐walled CVD system. The strips of Cu foil were electrochemically polished in phosphoric acid for 15 min to clean the bare Cu surface and were subsequently rinsed with distilled water followed by isopropyl alcohol. Then the Cu strips were loaded into a 4 in. quartz tube followed by evacuating the chamber to ≈3 mTorr; the temperature was then increased to 1000–1050 °C under an H_2_ environment (H_2_ flow rate of 5 sccm). After annealing of the Cu strips for 10 min, the graphene was synthesized by introducing methane gas (CH_4_ flow rate of 10 sccm) and then cooling under the same conditions. Following the growth, graphene layers were transferred onto SiO_2_/Si for further investigation using a conventional wet‐transfer method assisted by poly(methyl methacrylate) supporting layer and an aqueous solution of 1 m ammonium persulfate.

Afterward, the growth of WS_2_ was carried out on using a two‐zone furnace system with a WO_3_ thin film as the W source and S powder (99.998%, Sigma‐Aldrich) as the S source. For the uniform source, 1 nm of WO_3_ thin film was evenly deposited onto SiO_2_/Si using an e‐beam evaporator (WO_3_ pellets, Tae Won, 99.99%). The obtained graphene substrate was placed face‐to‐face on top of WO_3_ film. This WO_3_/Gr assembly was loaded at the center of the heat zone of the furnace, with S powder containing boat at the upstream of the furnace. Before heating, the whole furnace system was purged with 500 sccm Ar gas (99.999%). Then, 30 sccm Ar was introduced into the system as a carrier gas and the system was gradually heated up to 950 °C. After reaching 950 °C, S powder was started to melt (heated to 200 °C) using the equipped heating jacket and kept constant for whole growth process (40 min).

##### Structural Characterization

SEM was conducted in secondary electron (SE) and backscattered electron (BSE) modes using a cold field‐emission scanning electron microscope (Hitachi SU‐8220) operated at the accelerating voltage of 1 kV. The surface morphology and conductance of the APBs in WS_2_/graphene heterostructure were determined by an atomic force microscopy (Bruker Multimode 8) operated in tapping and contact modes, respectively. Conductive AFM (C‐AFM) measurements were carried out at a DC bias of 300 mV; bias was applied through conductive Pt–Ir coated tips with a normal resonance frequency of ≈13 kHz and a spring constant of ≈0.2 N m^−1^.

TEM observations were performed using a FEI Titan cube G2 60–300 transmission electron microscope (located at UNIST) equipped with image‐ and probe‐aberration correctors. Dark‐field mode was used for analyzing the exact orientation of WS_2_ and confirming the global features of APB. Atomic resolution TEM and STEM modes were used for atomic structure analysis of each type of APB. For TEM analysis, the WS_2_/graphene heterostructures grown on SiO_2_/Si substrates were directly transferred on to TEM grids using a wet‐transfer method. All TEM experiments were performed at the acceleration voltage of 80 kV to minimize beam damage.

##### Fabrication of MoS_2_ FETs with Ti/Au Contacts

To synthesize MoS_2_ FETs, MoS_2_ flakes were synthesized on silicon chip with 285 nm SiO_2_ using powder‐based CVD with a NaCl as a growth promoter. Prior to growth, 25 mg NaCl was dissolved in 10 mL distilled water for the 0.1 m NaCl promoter solution. A small droplet of 10 µL of promoter solution was pipetted on the corners of an additional SiO_2_ substrate. Afterward, baked out at 90 °C in order to remove the water completely. Two substrates, one for growth and the other with promoter, were loaded with the sputtered 5 nm‐thick MoO_3_ film face‐to‐face at the center of the quartz furnace, and 15 mg sulfur powder in alumina boats was placed in the upstream of the CVD system. After purging with 1000 sccm Ar flow, the CVD growth was then performed at atmospheric pressure with a continuous flow of 100 sccm Ar gas. The temperature of the furnace was gradually raised to 750 °C. When the temperature of the furnace approached ≈715 °C, S powder was started to melt (heated to 230 °C) using equipped heating jacket and remained for 10 min of the whole growth process.

Then, the MoS_2_ channel regions were defined by using e‐beam lithography (NBL, NB3) and Ar plasma etching. Next, the Ti/Au (10/80 nm) electrodes were contacted using the e‐beam lithography and e‐beam evaporator (Temescal FC‐2000). Thermally grown 300 nm thick SiO_2_ layer on heavily p‐doped Si was used as the back‐gate insulator. Any heat treatment after the fabrication was not conducted to avoid thermal budget effect. Electrical characterization was performed in a probe station (Lakeshore CRX‐4K) equipped with a Keithley 4200‐SCS detector at room temperature.

##### DFT Calculations

For DFT calculation, WS_2_ APB models were constructed in the form of zigzag and armchair nanoribbons (Figure S4, Supporting Information). Note that zigzag nanoribbons are used for W_rhomb_, W‐S‐W, S_rhomb_, and S‐W‐S models, while armchair nanoribbon was modeled for S_rhomb_ + W‐S‐W configuration. The optimized monolayer WS_2_ model is in good agreement with experimentally observed lattice parameters (i.e., *a* = *b* = 3.16 Å). The width of the optimized WS_2_ nanoribbon was ≈5 nm with APBs located at the center of the nanoribbon. Note that vacuum regions were introduced at about 30 Å in parallel and perpendicular directions to the zigzag WS_2_ nanoribbon (i.e., 80.0 × 3.2 × 29.5 Å^3^) and armchair WS_2_ nanoribbon (i.e., 5.5 × 80.0 × 29.5 Å^3^) to avoid the self‐interactions. All spin‐polarized DFT calculations were performed using DMol^3^ code^[^ ^38^, ^39^ ^]^ under generalized gradient approximation (GGA) with Perdew–Burke–Ernzerhof (PBE) functional.^[^ ^40^ ^]^ All electron core treatment including relativistic effect was adopted with DNP 4.4 level and real‐space cutoff of 4.9 Å. The smearing value was set to 0.005 Ha. The Monkhorst‐Pack scheme^[^ ^41^ ^]^ was used to sample the Brillouin‐zone as follows: 5 × 5 × 1 for monolayer WS_2_ unit cell, and 1 × 5 × 1 *k*‐points for WS_2_ zigzag nanoribbon, and 3 × 1 × 1 *k*‐points for WS_2_ armchair nanoribbon models, respectively. The convergence criteria for geometry optimizations of all considered model systems were set to 1.0 × 10^−5^ Ha, 0.002 Ha Å^−1^, and 0.005 Å, for energy, maximum force and maximum displacement, respectively. The reaction paths for structural transitions between S‐facing and W‐facing APBs were examined by transition state search calculation using linear and quadratic synchronous transit (LST/QST) method with the conjugated gradient (CG) refinement,^[^ ^42^ ^]^ where the RMS convergence criterion was set as 0.01 Ha Å.^−1^

## Conflict of Interest

The authors declare no conflict of interest.

## Supporting information

Supporting Information
