# Supplementary material for: Antiphase Boundaries as Faceted Metallic Wires in 2D Transition Metal Dichalcogenides
Source: Adv Sci (Weinh). 2020 Jun 8;7(15):2000788. doi: 10.1002/advs.202000788 (PMC7404160; doi:10.1002/advs.202000788)
Supplement: Supplementary file 1 — Supporting Information [file ADVS-7-2000788-s001.pdf]

© 2020 Wiley-VCH GmbH

ADVANCED SCIENCE
Open Access

Supporting Information

for *Adv. Sci.*, DOI: 10.1002/advs.202000788

Antiphase Boundaries as Faceted Metallic Wires in Two-dimensional Transition Metal Dichalcogenides

Jung Hwa Kim, Se-Yang Kim, Sung O Park, Gwan Yeong Jung, Seunguk Song, Ahrum Sohn, Sang-Woo Kim, Sang Kyu Kwak, Soon-Yong Kwon*, and Zonghoon Lee**

Supporting Information

Antiphase Boundaries as Faceted Metallic Wires in Two-dimensional Transition Metal Dichalcogenides

Jung Hwa Kim, Se-Yang Kim, Sung O Park, Gwan Yeong Jung, Seunguk Song, Ahrum Sohn, Sang-Woo Kim, Sang Kyu Kwak, Soon-Yong Kwon*, and Zonghoon Lee**

Dr. J. H. Kim, Dr. S.-Y. Kim, S. Song, Prof. S.-Y. Kwon, Prof. Z. Lee
School of Materials Science and Engineering, Ulsan National Institute of Science and Technology (UNIST), Ulsan 44919, Republic of Korea
E-mail: zhlee@unist.ac.kr

Dr. J. H. Kim, Prof. S. K. Kwak, Prof. Z. Lee
Center for Multidimensional Carbon Materials, Institute for Basic Science (IBS), Ulsan 44919, Republic of Korea

S. O Park, G. Y. Jung, Prof. S. K. Kwak
Department of Energy Engineering, School of Energy and Chemical Engineering, Ulsan National Institute of Science and Technology (UNIST), Ulsan 44919, Republic of Korea

Dr. A. Sohn, Prof. S.-W. Kim
School of Advanced Materials Science and Engineering, Sungkyunkwan University (SKKU), Suwon 16419, Republic of Korea

Dr. J. H. Kim, Dr. S.-Y. Kim, S. O Park contributed equally to this work.

Supplementary Note 1. Formation energy calculations

The formation energy of APBs (γ_{APB}) was calculated by the following equation,[1,2]

$$\gamma_{APB} + 2\gamma_{edge} = E_{slab} - n_W E_{WS_2} + (2n_W - n_S)\mu_S \quad (1)$$

where γ_{edge}, E_{slab}, n_W, E_{WS_2}, n_S, and μ_S, referred to the edge formation energy, total energy of WS_2 zigzag nanoribbon models, number of atoms of tungsten, total energy of monolayer WS_2 unit cell, number of atoms of sulfur, and chemical potential of sulfur, respectively. Note that the APB formation energy of S_{rhomb} + W-S-W model was calculated by the energy difference between the armchair WS_2 nanoribbon model including APB and its pristine nanoribbon form. The edge formation energy (γ_{edge}) was evaluated using triangular WS_2 flakes by following equations,

$$3(\gamma_{edge} + \gamma_{vertex}) = E_{flake} - n_W E_{WS_2} + (2n_W - n_S)\mu_S \quad (2)$$

where γ_{vertex} and E_{flake} referred to the formation energy of the vertex of the triangular WS_2 flake and total energy of the triangular WS_2 flake, respectively. Further details on the Equation (2) can be found in the earlier work by Schweiger *et al.*[3] For γ_{APB} and γ_{edge} calculations, the boundary of $\Delta\mu_S$ was set between –1.22 and 0 eV, where the formation of bulk WS_2 became thermodynamically spontaneous. In addition, we calculated total energy of each facet (E_{APB}) considering the fraction, facet length, and formation energy by the following equation,

$$E_{APB} = \sum_i (\alpha_i L_{APB}) \gamma_{APB,i} \quad (3)$$

where α_i, L_{APB}, and $\gamma_{APB,i}$ represent the fraction, total length of facet, and formation energy of each APB configuration *i*. For the straight model, the equation can be expressed by

$$E_{APB(\mathrm{str})} = L_{\mathrm{str}} \gamma_{APB,W\text{-}S\text{-}W} \quad (4)$$

where the APB is assumed to be ideally composed of W-S-W structure. Also, to qualitatively compare the total energies of each APB structure (*i.e.*, straight and faceted), we assumed the total APB length of straight model (L_{str}) to be 1 Å. For the faceted model, the APB structure was experimentally observed to be composed of 62% S_{rhomb} and 38% W-S-W APB configurations (Figure 4g). Note that these configurations were also thermodynamically more stable than S-W-S and W_{rhomb} structure for a whole range of sulfur chemical potential (Figure 4f). Accordingly, the total energy of faceted model can be defined as follows,

$$E_{APB(\mathrm{faceted})} = (\alpha_{S_{rhomb}} L_{\mathrm{facet}})\gamma_{APB,S_{rhomb}} + (\alpha_{W\text{-}S\text{-}W} L_{\mathrm{facet}})\gamma_{APB,W\text{-}S\text{-}W} \quad (5)$$

where $\alpha_{S_{rhomb}}$, $\alpha_{W\text{-}S\text{-}W}$, and L_{facet} represent the fraction of S_{rhomb}, fraction of W-S-W, and total length of faceted APB, respectively. Note that $\alpha_{S_{rhomb}}$ and $\alpha_{W\text{-}S\text{-}W}$ are set to be 0.62 and 0.38 while the L_{facet} was set to be 1.28 Å by the experiment, respectively.

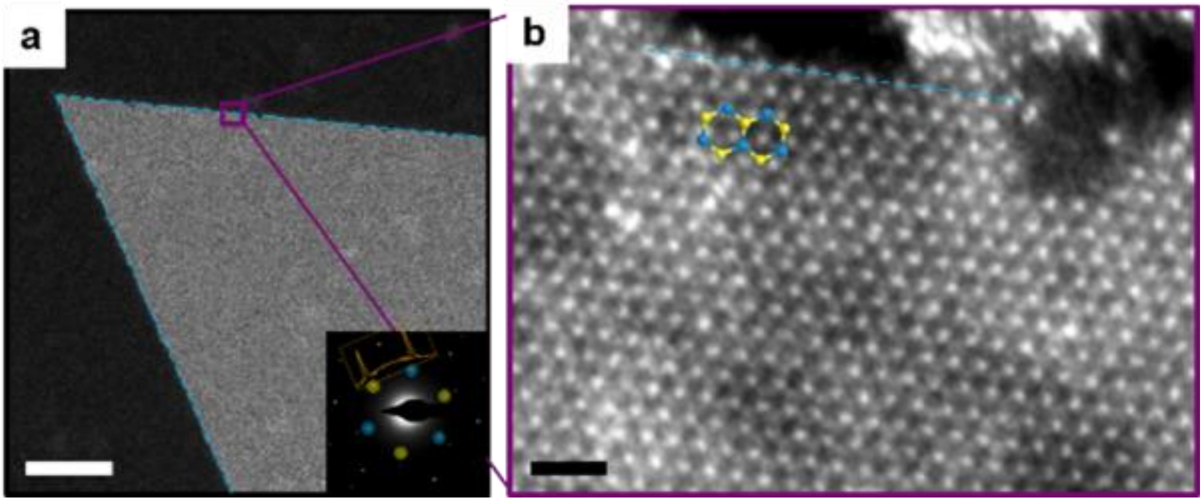

Supplementary Figure 1. W-terminated growth. (a) DF-TEM image and SAED pattern (inset) of WS_2 flake grown on the graphene substrate. (b) AR-STEM image of WS_2 edge. The blue dotted lines in (a,b) represent W-terminated edges. Scale bars for (a) and (b) are 100 nm and 1 nm, respectively.

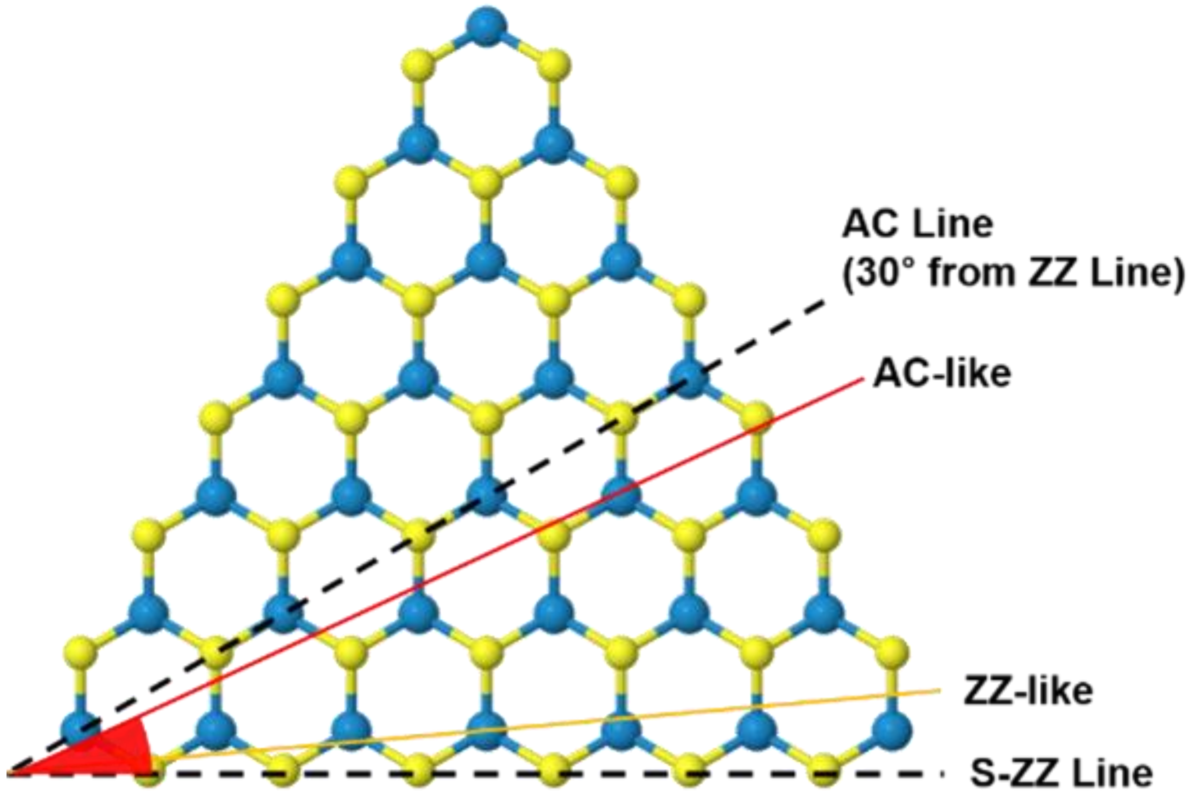

Supplementary Figure 2. Determination of APB angle with respect to the S-zigzag (S-ZZ) line. The armchair (AC) edge effect determines the APB angle by controlling kink density.

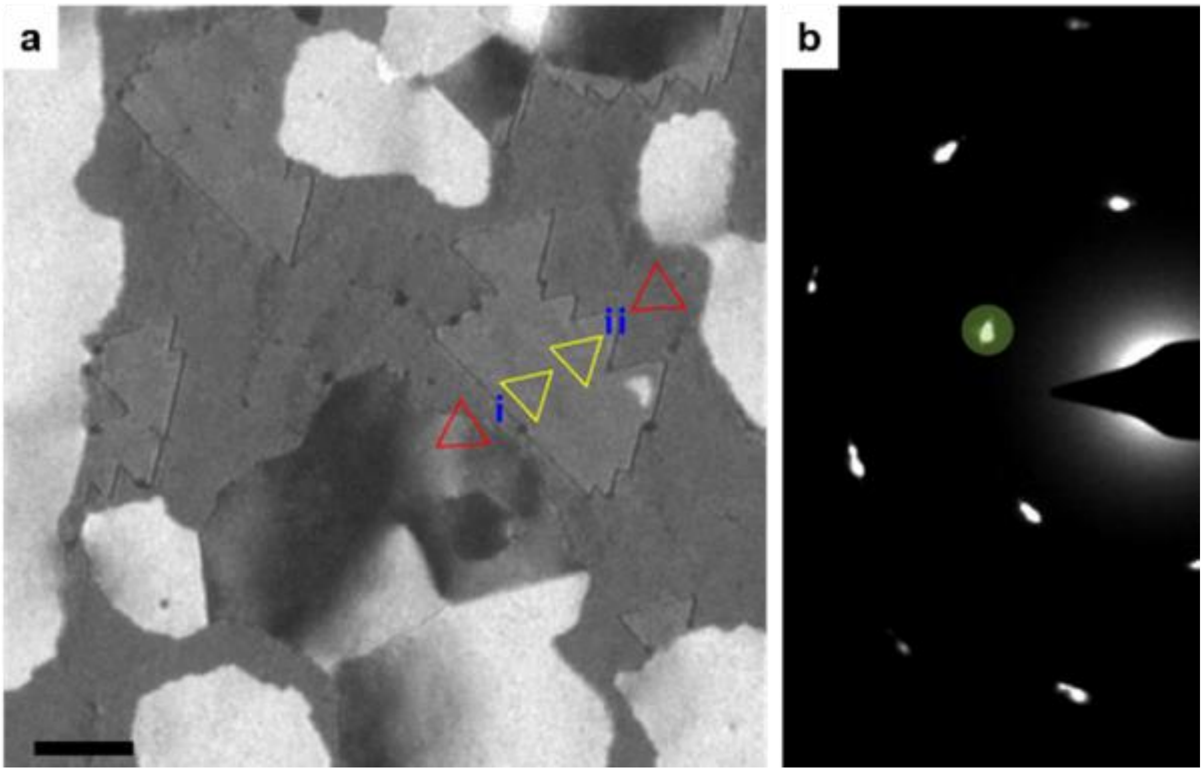

Supplementary Figure 3. APBs in MoS_2 grown on the sapphire substrate. (a) DF-TEM image and (b) the corresponding diffraction pattern. DF-TEM image was obtained by selecting the diffraction peak shaded by yellow as shown in (b). Triangles in (a) indicate Mo sublattice. When the two triangles meet via S-facing (case i), a linear APB is formed and for the Mo facing (case ii) APB has saw-toothed global structure. Scale bar, 50 nm.

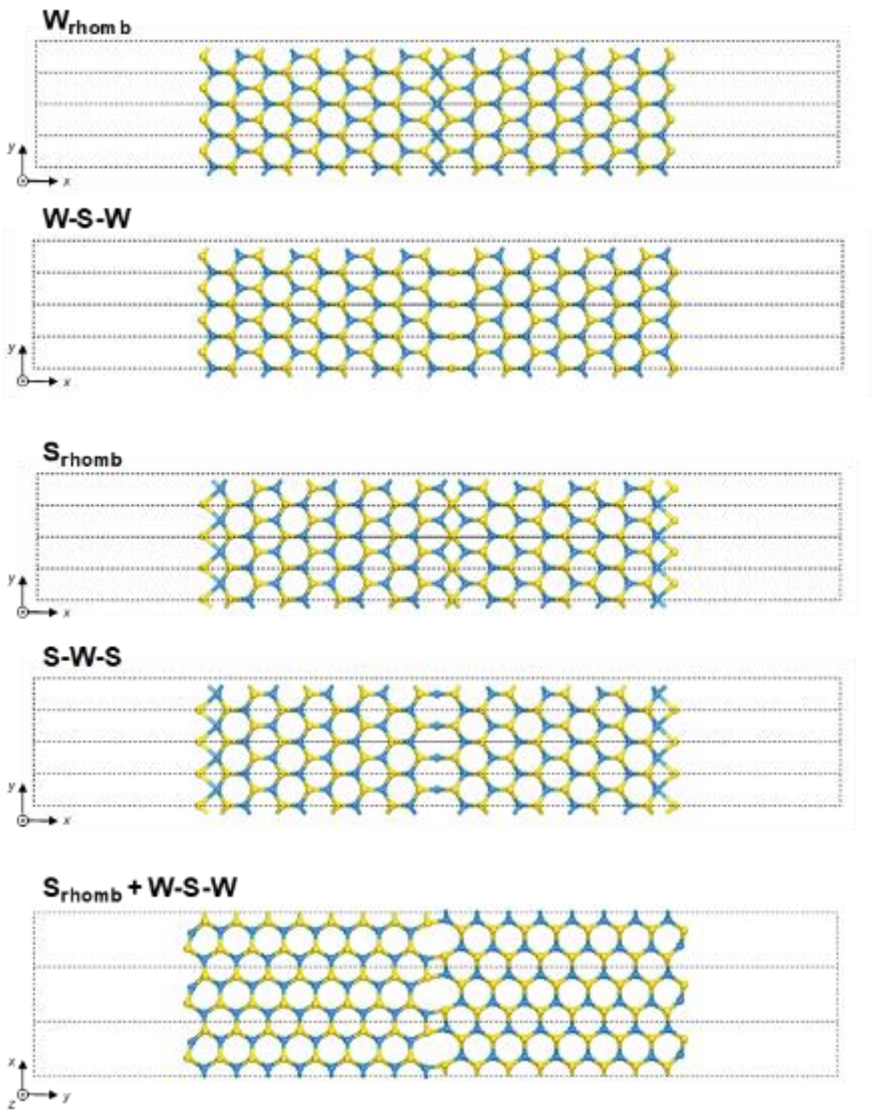

Supplementary Figure 4. Types of WS_2 APBs; ,'W$_{rhomb}$', 'W-S-W', 'S$_{rhomb}$', 'S-W-S', and 'S$_{rhomb}$ + W-S-W' APB configurations, where the S-edge and W-edge of zigzag WS_2 nanoribbons are terminated with 100% and 50% S coverage, respectively. Yellow and blue balls represent S and W atoms, respectively.

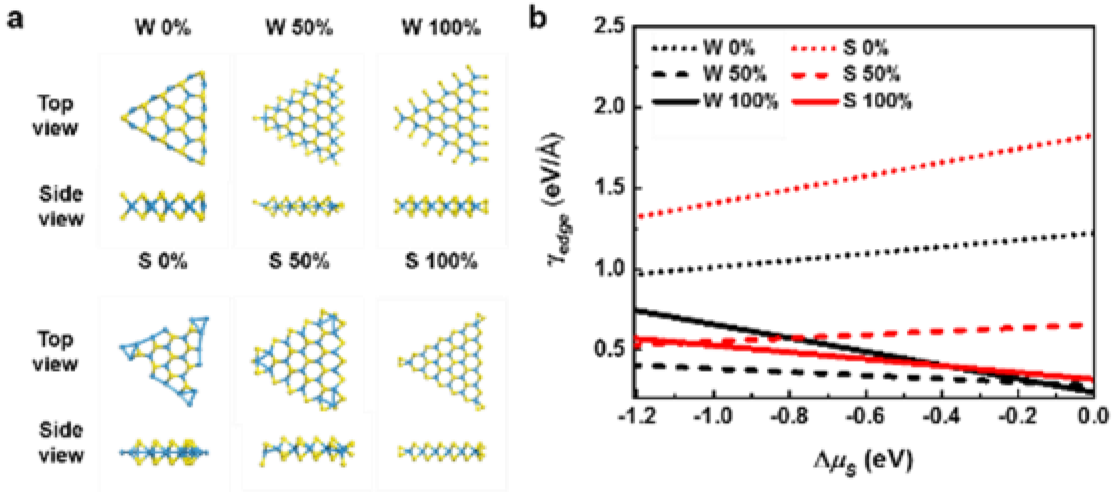

Supplementary Figure 5. Edge formation energy. (a) Optimized triangular WS_2 flakes consisted of 18 and 21 W atoms for the bulk region of W-edge and S-edge models, respectively. Note that 0%, 50%, and 100% coverages of S atoms are taken into account for the outermost edge configurations. (b) Edge formation energy normalized by length of the surface unit (γ_{edge}) as a function of chemical potential of S ($\Delta\mu_S$). WS_2 becomes stable within the range of -1.22 eV < $\Delta\mu_S$ < 0 eV where the upper and lower limits correspond to S-rich and S-poor conditions, respectively. Yellow and blue balls represent the S and W atoms, respectively.

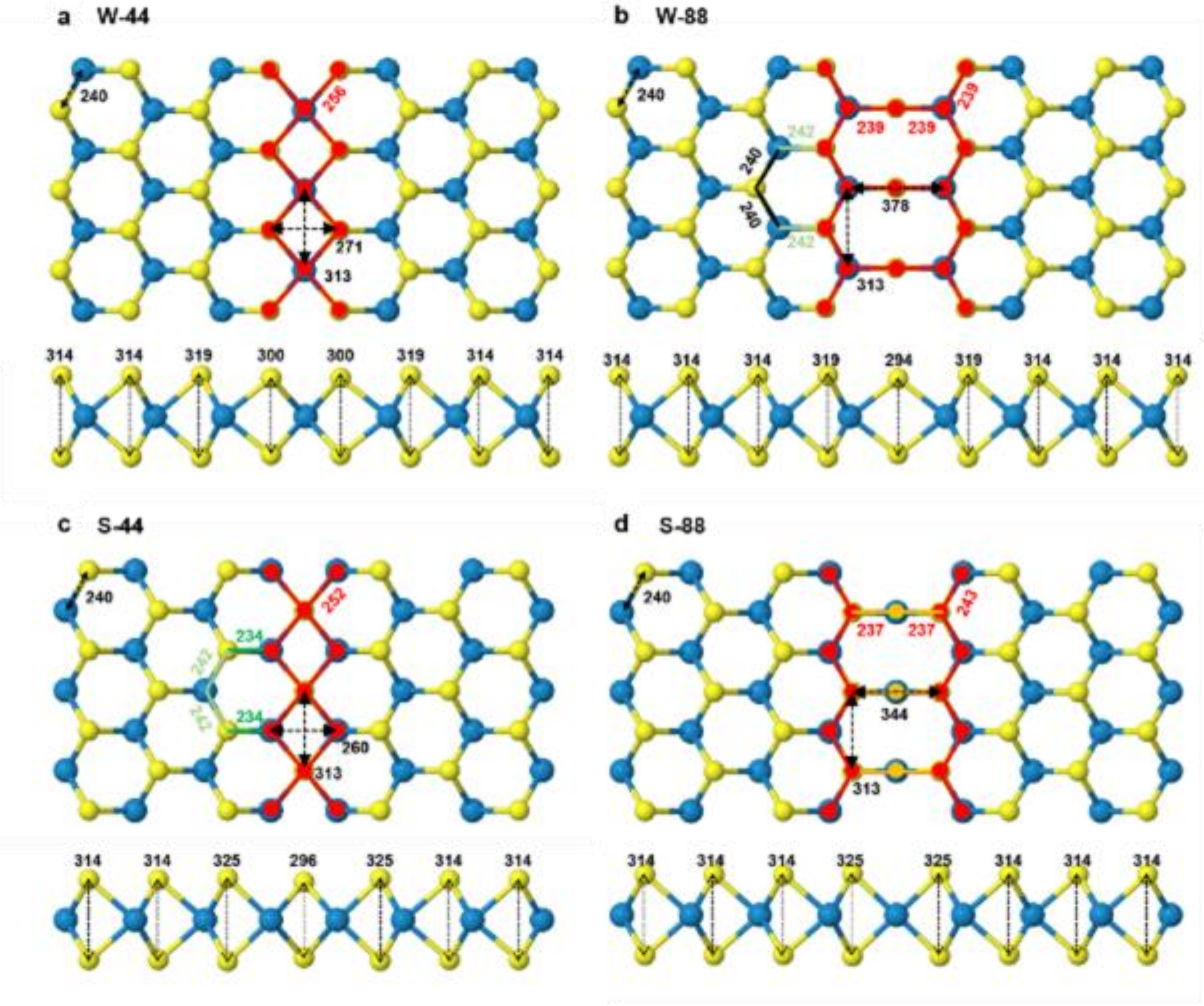

Supplementary Figure 6. Relaxed atomic configurations of (a) W-44, (b) W-88, (c) S-44, and (d) S-88 APBs. The numbers represent the length values and the unit is picometer (pm).

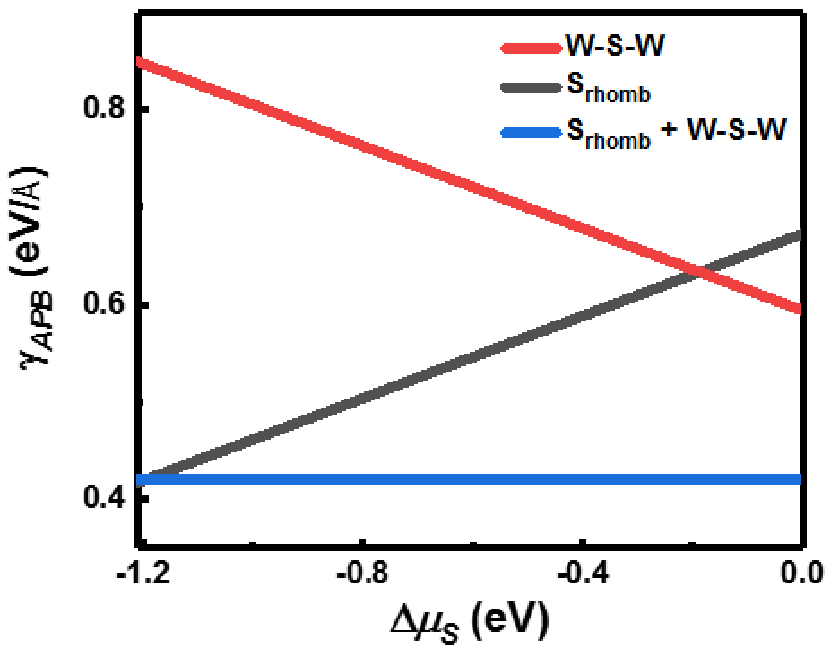

Supplementary Figure 7. APB formation energy (γ_{APB}) of 'W-S-W', 'S_{rhomb}', and 'S_{rhomb} + W-S-W' APBs as a function of chemical potential of S ($\Delta\mu_S$). WS_2 becomes stable within the range of -1.22 eV < $\Delta\mu_S$ < 0 eV where the upper and lower limits correspond to S-rich and S-poor conditions, respectively.

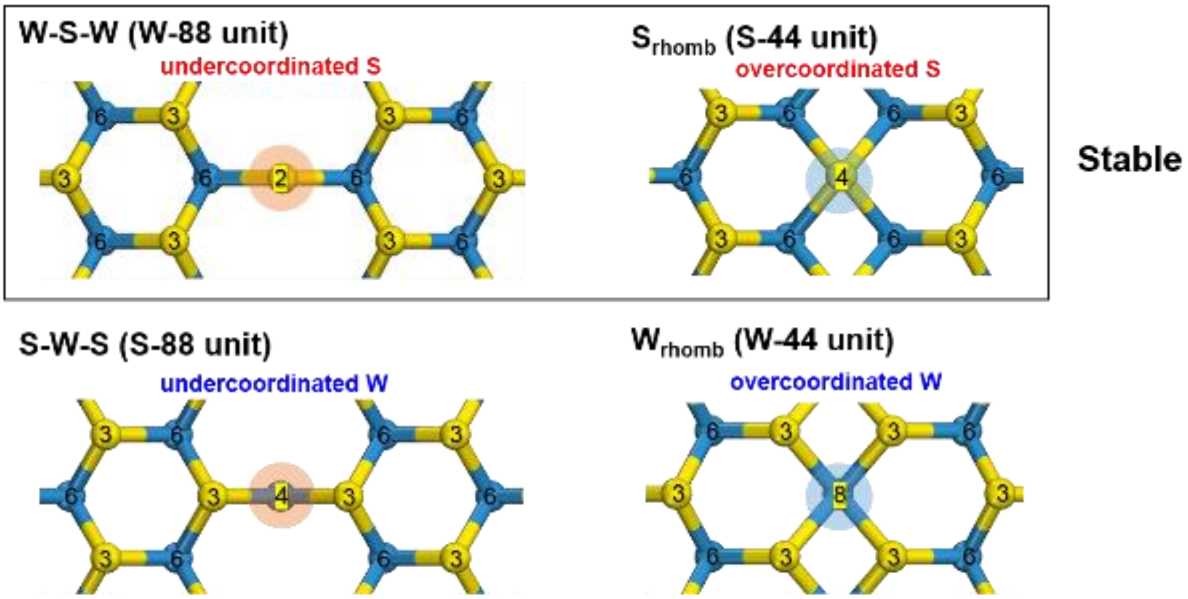

Supplementary Figure 8. Coordination numbers of S and W atoms consisting of W-S-W, S_{rhomb}, S-W-S, and W_{rhomb} APBs. Yellow and blue balls represent S and W atoms, respectively. Red or blue circles indicate under-coordinated or over-coordinated atoms compared to the bulk ones.

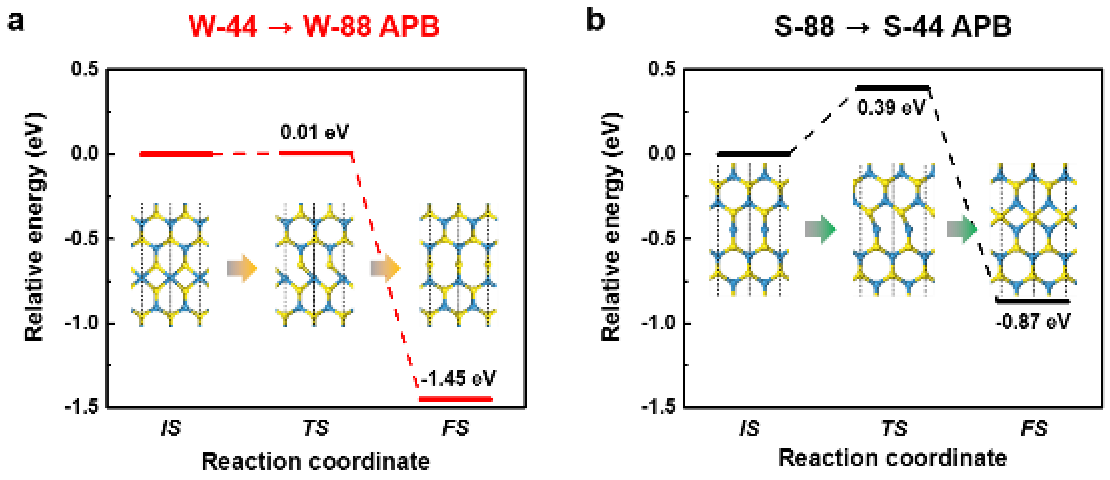

Supplementary Figure 9. Energy diagrams of structural transition between (a) W-facing and (b) S-facing APBs. Insets indicate the atomic configurations for the initial state (*IS*) (*i.e.*, W-44 for W-facing and S-88 for S-facing), transition state (*TS*), and final state (*FS*) (*i.e.*, W-88 for W-facing and S-44 for S-facing) of the reaction mechanism. Yellow and blue balls represent S and W atoms, respectively.

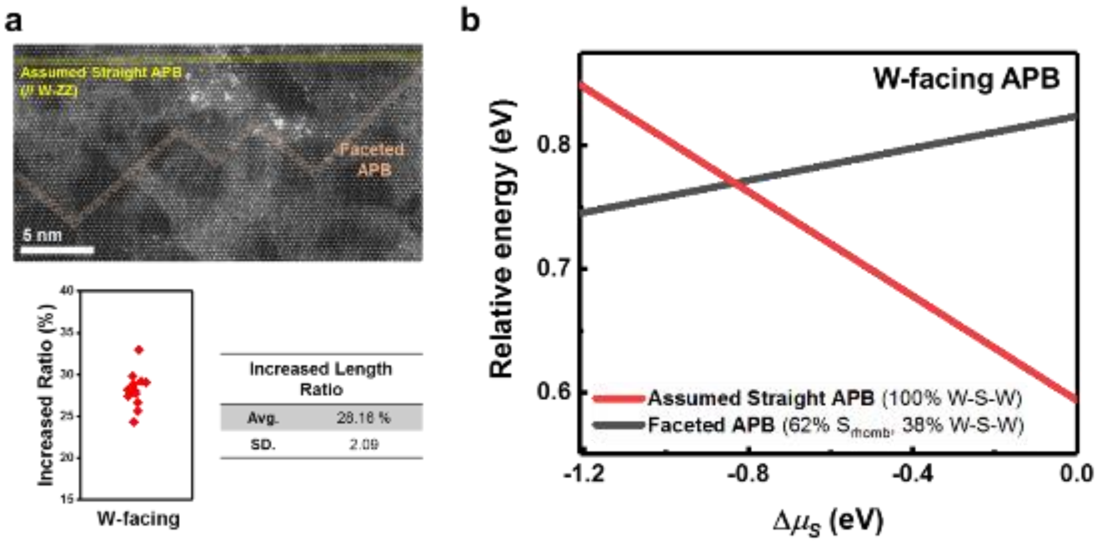

Supplementary Figure 10. Total energy calculation of faceted APB. (a) Faceted-morphology-driven increased APB length ratio compared to the expected straight APB along W-ZZ. (b) Relative energy of W-facing APB structure in terms of the structure (*i.e.*, straight and faceted structures). Note that the APB formation energy obtained from zigzag WS_2 nanoribbon models were only considered in the calculation to simplify the model system. The facet length of straight APB was assumed to be 1 Å.

Our result shows that the total energy of faceted APB becomes lower than that of the assumed straight APB when $\Delta\mu_S$ is lower than -0.83 (i.e., S-poor condition). Following the previous study,[4] WS_2 flakes of which edges were terminated by tungsten were synthesized under S-poor condition, which are consistent with our experimental observation (Figure 1a and Supplementary Figure 1).

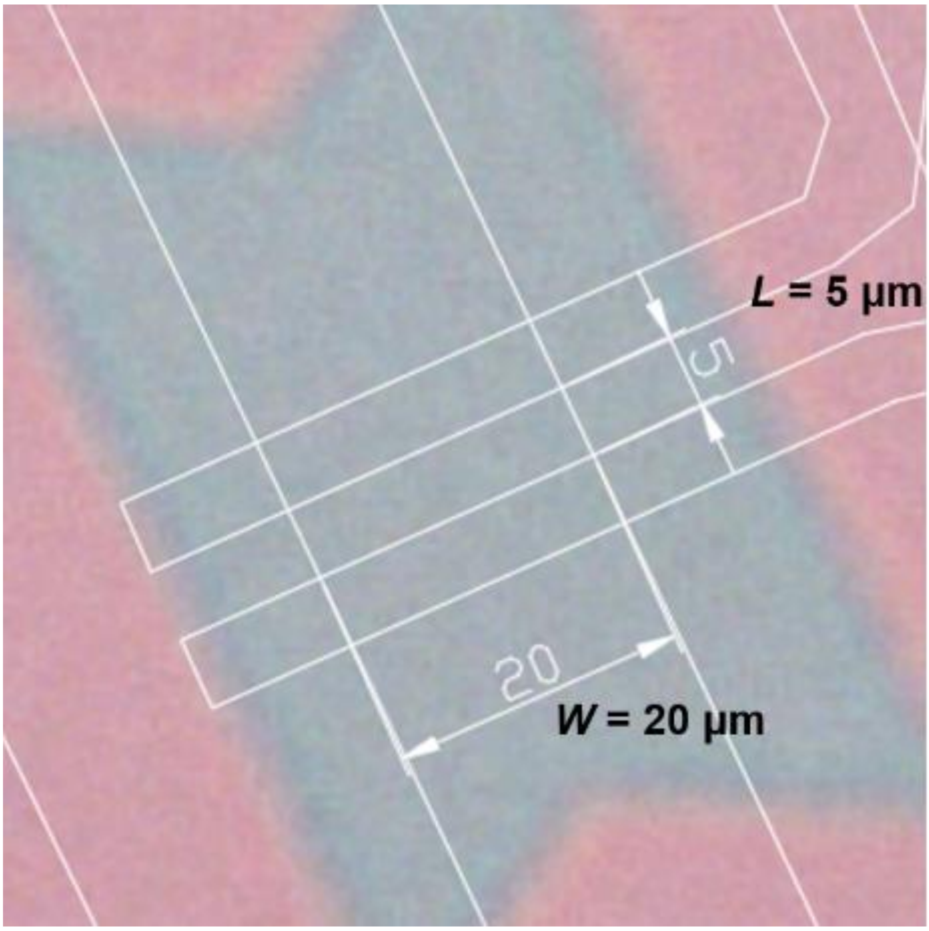

Supplementary Figure 11. MoS_2 field-effect transistor (FET) device design. Channel width and length are fixed to the 20 μm and 5 μm, respectively. Scale bar, 5 μm.

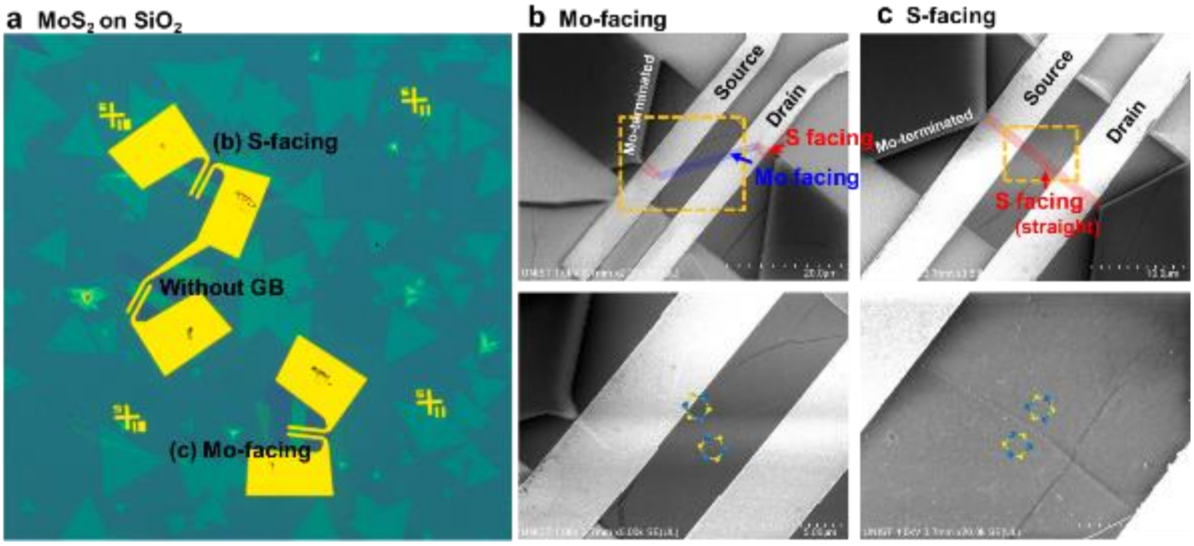

Supplementary Figure 12. MoS_2 FET device to illustrate the effect of facet in TMDs' APBs. (a) Optical microscopy image of FET with channel width and length normalization. (b,c) SEM images of FET devices with Mo-facing and S-facing APBs, respectively. SEM images were obtained using secondary electron (SE) mode detector. APBs can be observed, though not very clearly.

Note that while symmetric ribbon-shaped flakes have S-facing APB exclusively (Supplementary Figure 12c), the asymmetrically merged flakes have both S-facing and Mo-facing APB with zigzag alignment (Supplementary Figure 12b). Because the contributions both types of APB make APB irregular, we have difficulty in identifying exact morphology of APB lines in macroscopic scale. Therefore, we fabricated the devices which have the electrodes across the symmetrically- and asymmetrically-merged grains to investigate the effect of faceted APB on electrical properties.

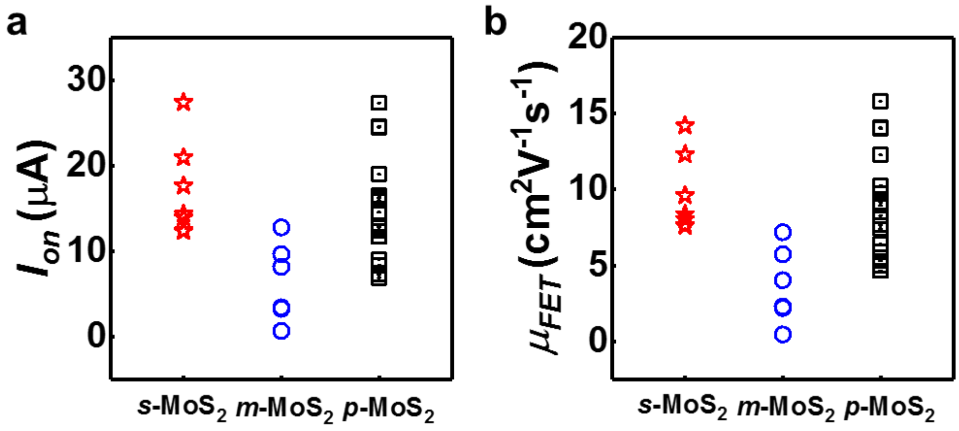

Supplementary Figure 13. I_{on} and μ_{FET} of MoS_2 devices with S-facing (s-MoS_2), Mo-facing APB (m-MoS_2) and without APB (p-MoS_2).

		S-facing	W-facing
Facet Length (nm)	Average	155.892	128.725
	S.D.	18.195	11.146
Inclination Angle (degree)	Average	14.889	22.421
	S.D.	6.071	3.636

Supplementary Table 1. Averaged facet length and inclination angle (from S-ZZ) of S-facing and W-facing APB.

	s-MoS_2	*m*-MoS_2	*p*-MoS_2
I_{on} [μA]	17.01 ± 5.5	6.35 ± 4.6	14.9 ± 6.1
μ_{FET} [$cm^2V^{-1}s^{-1}$]	9.7 ± 2.6	3.7 ± 2.5	9.1 ± 3.5
V_{th} [V]	12.4 ± 3.3	14.6 ± 5.3	15.2 ± 4.1

Supplementary Table 2. Summarized transport properties of *s*-MoS_2, *m*-MoS_2, and *p*-MoS_2 devices.

Supplementary References

[1] M. V. Bollinger, K. W. Jacobsen, J. K. Norskov, *Phys. Rev. B* **2003**, 67, 085410.

[2] X. L. Zou, B. I. Yakobson, *Small* **2015**, 11, 4503.

[3] H. Schweiger, P. Raybaud, G. Kresse, H. Toulhoat, *J. Catal.* **2002**, 207, 76.

[4] S. Wang, Y. Rong, Y. Fan, M. Pacios, H. Bhaskaran, K. He, J. H. Warner, *Chem. Mater.* **2014**, 26, 6371.
